# Redox and metabolic regulation of transcription

**DOI:** 10.18632/oncotarget.13459

**Published:** 2016-11-18

**Authors:** Glenn Marsboom, Jalees Rehman

**Affiliations:** Department of Pharmacology and Department of Medicine, Division of Cardiology, University of Illinois College of Medicine, Chicago, IL, USA

**Keywords:** reactive oxygen species, redox state, metabolism, cancer cells, transcription factor regulation

Reactive oxygen species (ROS) production and the resultant shift in the cellular redox state can exert a biphasic effect on cellular functions such as survival, migration or proliferation. At high levels, ROS can induce broad DNA and protein damage which can result in cell death, but at lower levels ROS act as signaling molecules that sustain proliferation or activate specific stress-response pathways. The molecular mechanisms underlying the activation of specific signaling pathways and transcriptional programs are not fully understood but one key mechanism is the oxidation of critical cysteine residues in transcription factors which in turn affect DNA binding and transcriptional activity.

Redox regulated transcription factors have been implicated in carcinogenesis and tumor progression. The NRF2 transcription factor regulates antioxidant defenses and is sequestered in the cytoplasm by KEAP1, but oxidation of cysteines in KEAP1 leads to the release of NRF2 and the induction of antioxidant and cytoprotective enzymes which enhances chemoresistance of cancer cells [1]. Transcription factors which directly regulate tumor growth are also regulated by the cellular redox state. The 3-dimensional structure and DNA binding properties of the p53 tumor suppressor protein depend on the reduced state of several cysteines [2] while the DNA binding of the proto-oncogenes c-FOS/c-JUN also depends on a single cysteine in each protein [3].

Our recent work demonstrates that the pluripotency transcription factor OCT4 is also directly regulated by the cellular redox state [4]. In human embryonic stem cells (ESCs), oxidation of cysteines leads to reduced DNA binding and subsequent rapid degradation of the OCT4 protein. Importantly, suppressing cellular glutamine metabolism similarly results in OCT4 degradation. Because glutathione and NADPH production, key determinants of redox homeostasis, are dependent on glutamine metabolism, our findings highlight a mechanism by which metabolic cues can affect pluripotency and the differentiation state of stem cells.

Glutamine metabolism is upregulated in both ESCs and certain types of cancers [5, 6]. In addition to regulating the intracellular redox status, it also provides cells with carbon and nitrogen atoms for biosynthesis and supports energy generation. Epigenetic modifications of histones are indirectly dependent on glutamine metabolism as well [5]. The “addiction” of certain cancer cells to glutamine as a carbon source is thought to enhance metabolic flexibility and resilience of malignant cells. Activation of the proto-oncogene c-MYC leads to an upregulation of glutaminase, the enzyme involved in conversion of glutamine into glutamate, and cancers with c-MYC activation are exquisitely sensitive to inhibition of glutamine metabolism [6]. However, the molecular effects of targeting glutamine metabolism in stem cells or cancer cells have not been fully understood.

Our findings demonstrate that the glutamine availability affects the cellular redox state and in turn regulates transcription factors such as OCT4. Suppression of glutamine metabolism could reduce pluripotency of stem cells and possibly cancer stem cells -although the role of pluripotency transcription factors in malignancies remains controversial- and other redox-sensitive transcription factors that are critical for tumor formation and progression such as NRF2 and p53 could be affected as well.

A major unanswered question is how oxidation/reduction of individual transcription factor cysteine residues is regulated. Cysteine oxidation depends on the local electrostatic environment, with positively charged amino acids like arginine or lysine enhancing reactivity. Therefore, even within the same protein, not all cysteines have the same reactivity. Cysteine oxidation is also readily reversible, for example by thioredoxins. A recent proteomic paper has shown that Thioredoxin-1 specifically interacts with a number of oxidized proteins. In lungs exposed to hyperoxia, a method to induce ROS, a total of 17 Thioredoxin-1 interacting proteins were identified [7]. This finding suggests that reduction of cysteines might not be a random event, but that certain proteins are preferably kept in a reduced state. Further research is needed to understand the oxidation state of transcription factors implicated in tumor cells.

Transcription factors that regulate stem cell function and tumor growth can act as sensors for shifts in cellular metabolism and the redox state. This highlights the complex interplay of metabolic and redox pathways but also provides novel mechanistic insights and therapeutic targets in stem cells or tumor cells.
